# Supplementary material for: Indirect Genetic Effects of ADIPOQ Variants on Lipid Levels in a Sibling Study of a Rural Chinese Population
Source: Genes (Basel). 2022 Jan 17;13(1):161. doi: 10.3390/genes13010161 (PMC8774628; doi:10.3390/genes13010161)
Supplement: Supplementary file 1 [file genes-13-00161-s001.zip › genes-1529505-Supplementary materials.pdf]

Table S1. SNPs selected for the study.

SNP	Position	Max	Min	Missing rate	MAF	P for HWE
rs16861194	3: 186841636	A	G	0	0.172	0.033
rs266729	3: 186841685	C	G	0	0.283	0.268
rs182052	3: 186842993	A	G	0	0.482	0.152

Table S2. Phenotypic correlations between sibling pairs.

Variables	r	*P*
BMI	0.218	<0.001
WC	0.218	<0.001
SBP	0.201	<0.001
DBP	0.249	<0.001
FBG	0.224	<0.001
TG	0.200	<0.001
TC	0.620	<0.001
HDL-C	0.670	<0.001
LDL-C	0.553	<0.001

Table S3. Phenotypic correlations between sibling pairs stratified by sex.

Variables	Same-sex siblings		Opposite-sex siblings	
	r	*P*	r	*P*
TG	0.226	<0.001	0.158	<0.001
TC	0.625	<0.001	0.611	<0.001
HDL-C	0.655	<0.001	0.697	<0.001
LDL-C	0.559	<0.001	0.544	<0.001

Table S4. Phenotypic correlations between sibling pairs stratified by genotype and sex.

Sex	Variables	AA/AG-genotype siblings		GG genotype siblings		Opposite-genotype siblings	
		r	P	r	P	r	P
All	TG	0.161	<0.001	0.365	<0.001	0.244	<0.001
	TC	0.607	<0.001	0.661	<0.001	0.628	<0.001
	HDL-C	0.686	<0.001	0.624	<0.001	0.660	<0.001
	LDL-C	0.541	<0.001	0.601	<0.001	0.551	<0.001
Same-sex siblings	TG	0.184	<0.001	0.412	<0.001	0.275	<0.001
	TC	0.624	<0.001	0.654	<0.001	0.613	<0.001
	HDL-C	0.687	<0.001	0.581	<0.001	0.625	<0.001
	LDL-C	0.555	<0.001	0.578	<0.001	0.564	<0.001
Opposite-sex siblings	TG	0.125	0.011	0.292	0.011	0.200	0.012
	TC	0.578	<0.001	0.671	<0.001	0.653	<0.001
	HDL-C	0.683	<0.001	0.713	<0.001	0.713	<0.001
	LDL-C	0.518	<0.001	0.630	<0.001	0.539	<0.001
